# Supplementary figures and images for: Bacterial exonuclease III expands its enzymatic activities on single-stranded DNA
Source: eLife. 2024 Jul 3;13:RP95648. doi: 10.7554/eLife.95648 (PMC11221836; doi:10.7554/eLife.95648)

M D-T7 exo  
T7 exo  
D-APE1  
APE1  
D-ExoIII  
ExoIII  
D-Cas12a  
Cas12a

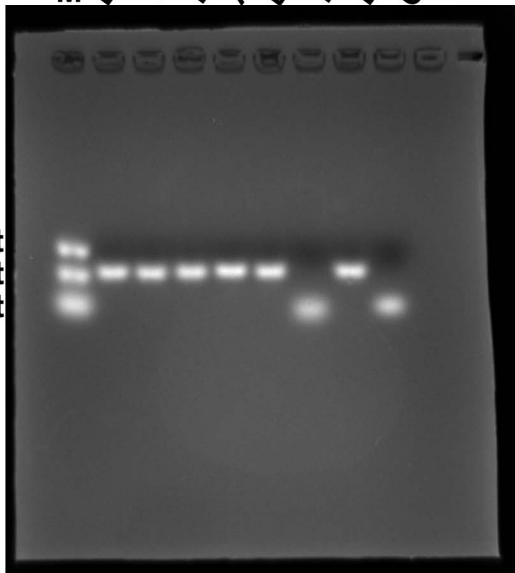

Supplement: Figure 2—source data 1. [file elife-95648-fig2-data1.zip › Figure 2-Source Data 1/Figure 2B-labelled.pdf]

**M 0 1 2 5 10 15 (Min)**

**30 nt**  
**15 nt**  
**5 nt**

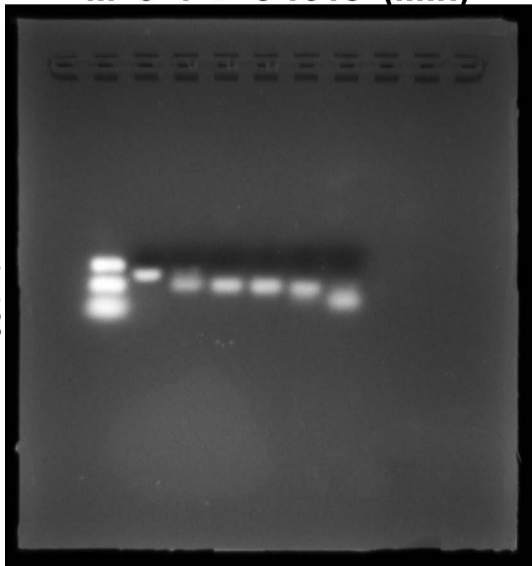

Supplement: Figure 2—source data 1. [file elife-95648-fig2-data1.zip › Figure 2-Source Data 1/Figure 2D-labelled.pdf]

U E E E E E E E E E

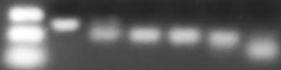

Supplement: Figure 2—source data 1. [file elife-95648-fig2-data1.zip › Figure 2-Source Data 1/Figure 2D-raw.pdf]

M D-T7 exo T7 exo D-APE1 APE1 D-ExoIII ExoIII D-Cas12a Cas12a

30 nt  
15 nt  
5 nt

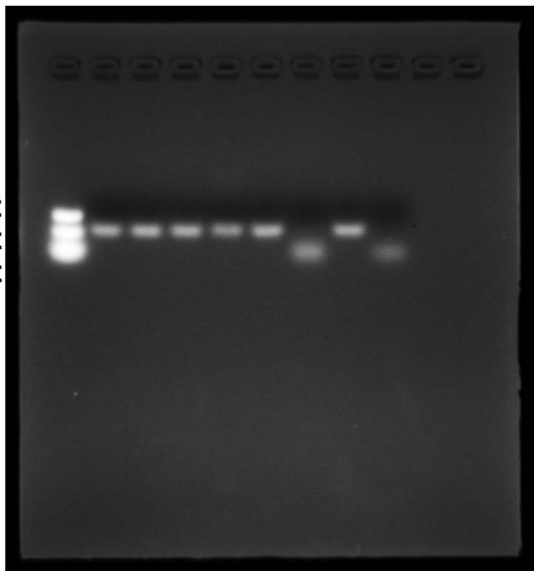

Supplement: Figure 2—source data 1. [file elife-95648-fig2-data1.zip › Figure 2-Source Data 1/Figure 2F-labelled.pdf]

**M 0 1 2 5 10 15 (Min)**

**30 nt**  
**15 nt**  
**5 nt**

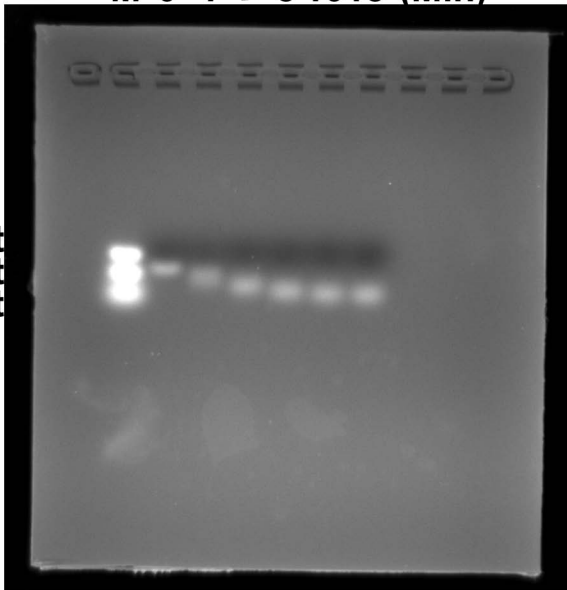

Supplement: Figure 2—source data 1. [file elife-95648-fig2-data1.zip › Figure 2-Source Data 1/Figure 2H-labelled.pdf]

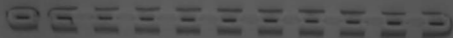

Supplement: Figure 2—source data 1. [file elife-95648-fig2-data1.zip › Figure 2-Source Data 1/Figure 2H-raw.pdf]

M D-T7 exo  
T7 exo D-APE1  
APE1 D-ExoIII  
ExoIII D-Cas12a  
Cas12a

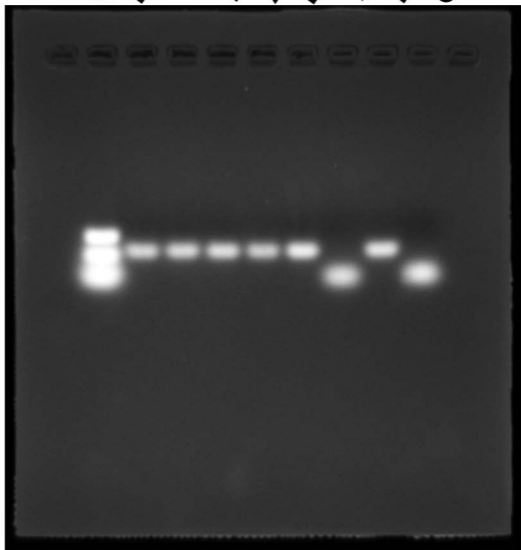

30 nt  
15 nt  
5 nt

Supplement: Figure 2—source data 1. [file elife-95648-fig2-data1.zip › Figure 2-Source Data 1/Figure 2J-labelled.pdf]

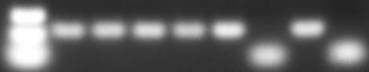

Supplement: Figure 2—source data 1. [file elife-95648-fig2-data1.zip › Figure 2-Source Data 1/Figure 2J-raw.pdf]

**M 0 1 2 5 10 15 (Min)**

**30 nt**  
**15 nt**  
**5 nt**

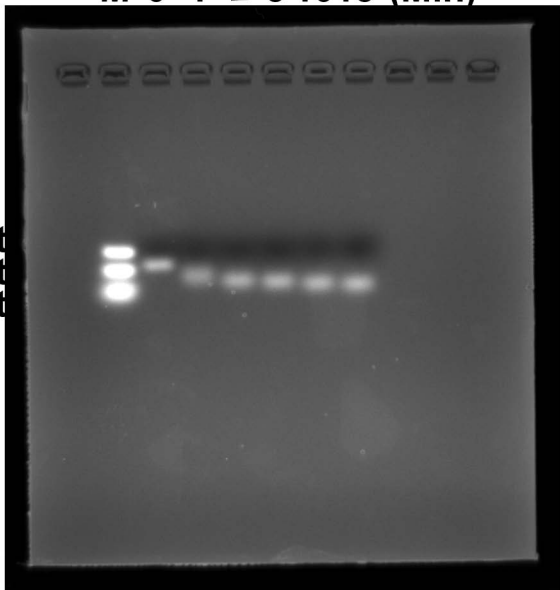

Supplement: Figure 2—source data 1. [file elife-95648-fig2-data1.zip › Figure 2-Source Data 1/Figure 2L-labelled.pdf]

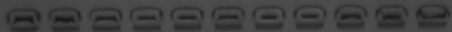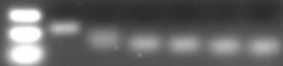

Supplement: Figure 2—source data 1. [file elife-95648-fig2-data1.zip › Figure 2-Source Data 1/Figure 2L-raw.pdf]

ssDNA

dsDNA

Time (Min)

0 1 2 5 10

0 1 2 5 10

5nt

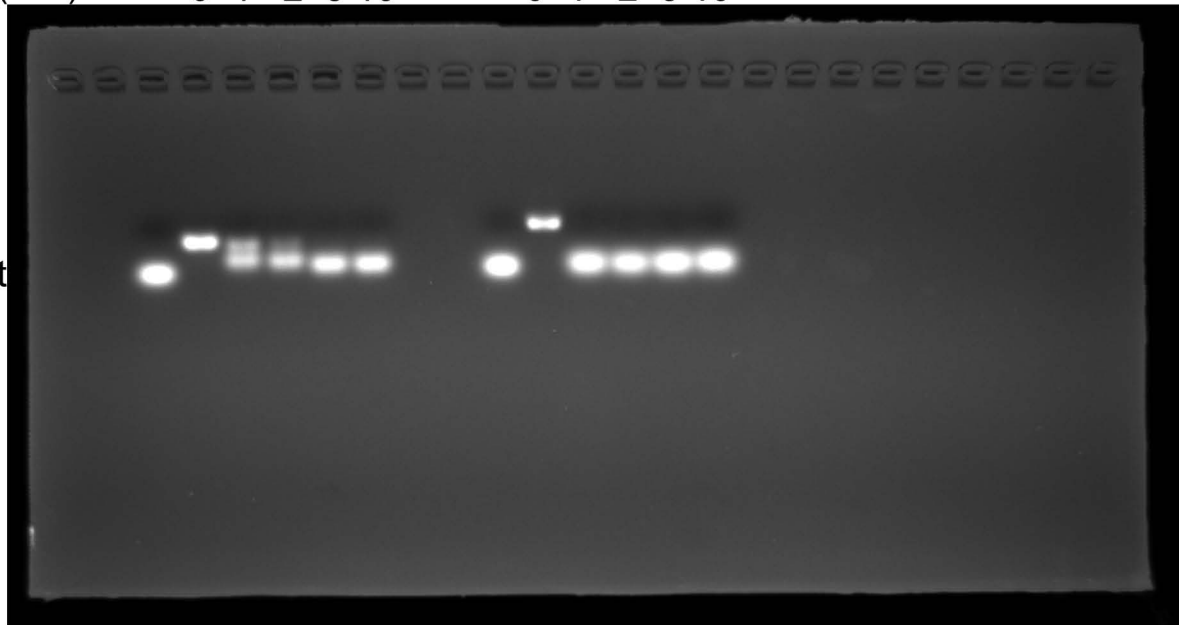

Supplement: Figure 2—figure supplement 1—source data 1. [file elife-95648-fig2-figsupp1-data1.zip › Figure 2-figure supplement 1-Souce data/Figure 2-figure supplement 1-labelled.pdf]

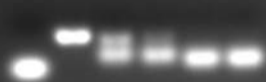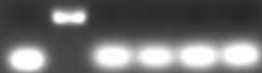

Supplement: Figure 2—figure supplement 1—source data 1. [file elife-95648-fig2-figsupp1-data1.zip › Figure 2-figure supplement 1-Souce data/Figure 2-figure supplement 1-raw.pdf]

## Probe 1

ExoIII (10 U/ $\mu$ l)

- + +

Mg<sup>2+</sup> (1 mM)

+ + +

EDTA (10 mM)

+ + -

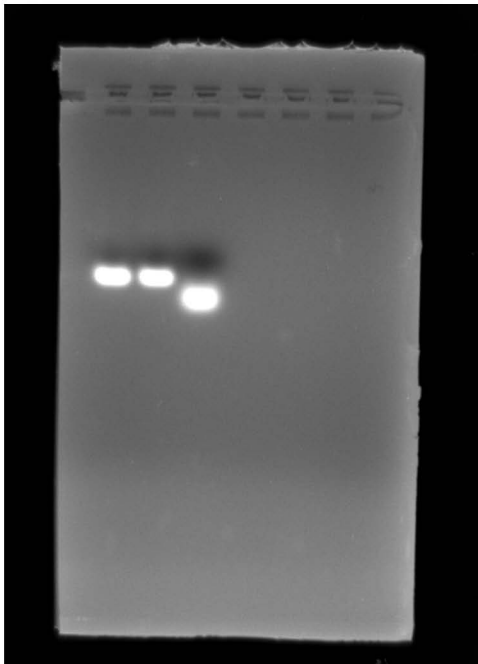

Supplement: Figure 4—source data 1. [file elife-95648-fig4-data1.zip › Figure 4/Figure 4B-Probe 1-labelled.pdf]

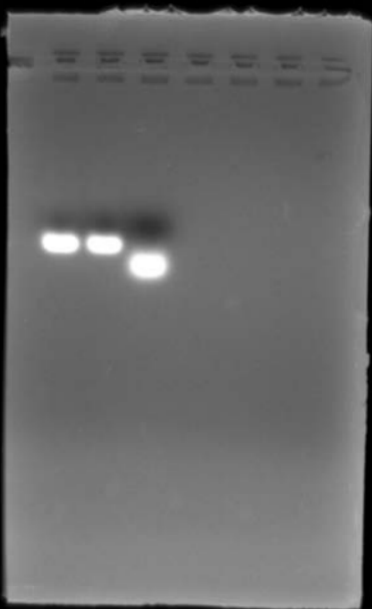

Supplement: Figure 4—source data 1. [file elife-95648-fig4-data1.zip › Figure 4/Figure 4B-Probe 1-raw.pdf]

## Probe 2

ExoIII (10 U/ $\mu$ l)

- + +

Mg<sup>2+</sup> (1 mM)

+ + +

EDTA (10 mM)

+ + -

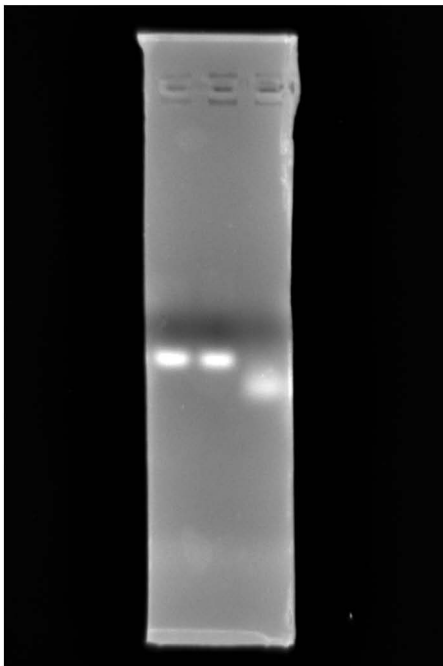

Supplement: Figure 4—source data 1. [file elife-95648-fig4-data1.zip › Figure 4/Figure 4B-Probe 2-labelled.pdf]

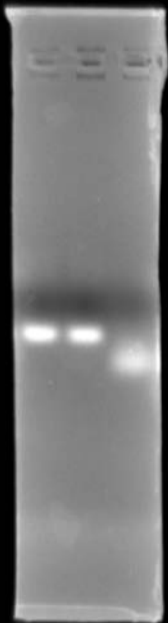

Supplement: Figure 4—source data 1. [file elife-95648-fig4-data1.zip › Figure 4/Figure 4B-Probe 2-raw.pdf]

## Probe 3

ExoIII (10 U/ $\mu$ l)

Mg<sup>2+</sup> (1 mM)

EDTA (10 mM)

- + +

+ + +

+ + -

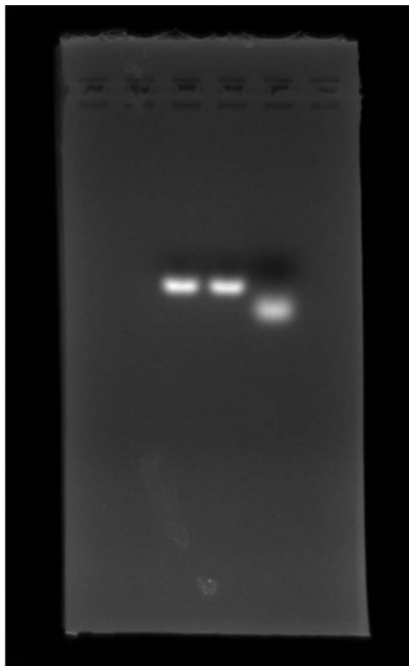

Supplement: Figure 4—source data 1. [file elife-95648-fig4-data1.zip › Figure 4/Figure 4B-Probe 3-labelled.pdf]

Probe 3 (0.5  $\mu\text{M}$ )

ExoIII      0   0.025   0.125   0.250   0.500   1.250   ( $\mu\text{M}$ )

20 nt

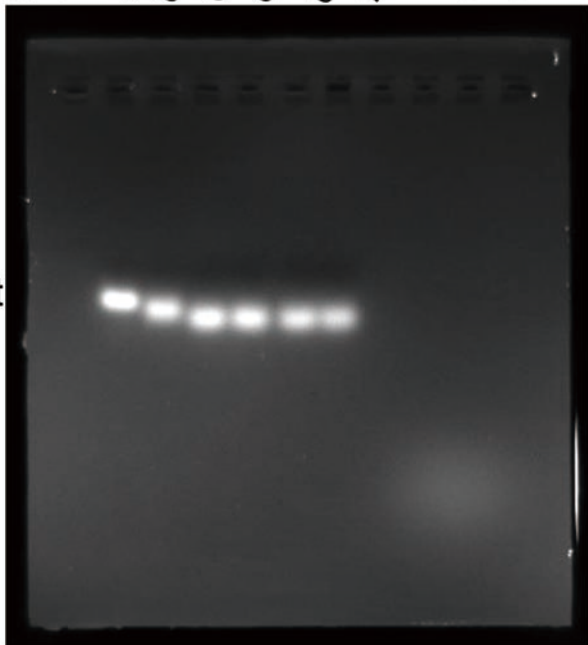

Supplement: Figure 4—source data 1. [file elife-95648-fig4-data1.zip › Figure 4/Figure 4C-labelled.pdf]

1000

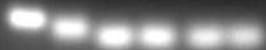

Supplement: Figure 4—source data 1. [file elife-95648-fig4-data1.zip › Figure 4/Figure 4C-raw.pdf]

# Probe 3 (0.5 $\mu$ M)

T ( $^{\circ}$ C)   NT   0   16   25   37   42   50

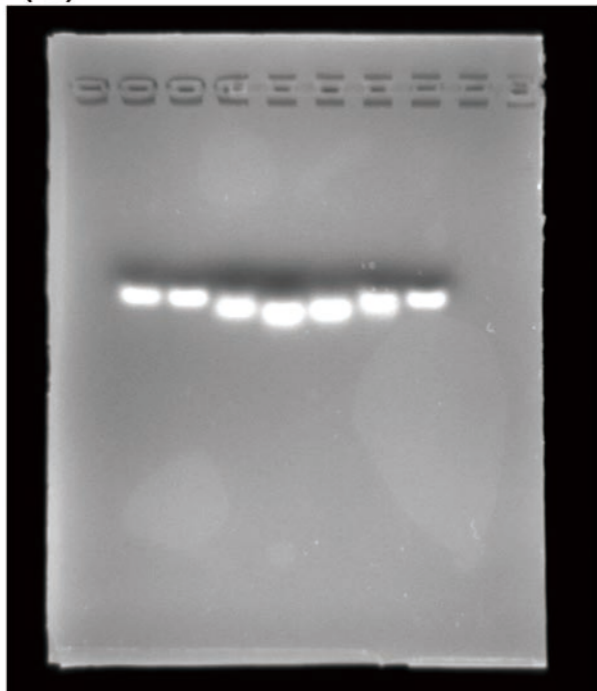

Supplement: Figure 4—source data 1. [file elife-95648-fig4-data1.zip › Figure 4/Figure 4E-labelled.pdf]

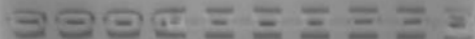

Supplement: Figure 4—source data 1. [file elife-95648-fig4-data1.zip › Figure 4/Figure 4E-raw.pdf]

$A_{20}$   $C_{20}$   $T_{20}$   
M 0 5 15 0 5 15 0 5 15

30 nt  
20 nt  
5 nt

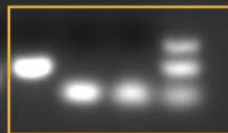

Unrelated bands

Supplement: Figure 4—source data 1. [file elife-95648-fig4-data1.zip › Figure 4/Figure 4G-labelled.pdf]

100 200 300 400 500 600 700 800 900 1000 1100 1200 1300 1400 1500 1600 1700 1800 1900 2000 2100 2200 2300 2400 2500 2600 2700 2800 2900 3000 3100 3200 3300 3400 3500 3600 3700 3800 3900 4000 4100 4200 4300 4400 4500 4600 4700 4800 4900 5000 5100 5200 5300 5400 5500 5600 5700 5800 5900 6000 6100 6200 6300 6400 6500 6600 6700 6800 6900 7000 7100 7200 7300 7400 7500 7600 7700 7800 7900 8000 8100 8200 8300 8400 8500 8600 8700 8800 8900 9000 9100 9200 9300 9400 9500 9600 9700 9800 9900 10000

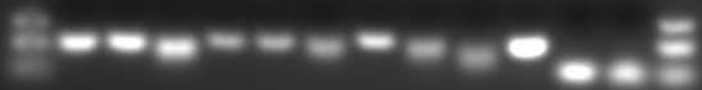

Supplement: Figure 4—source data 1. [file elife-95648-fig4-data1.zip › Figure 4/Figure 4G-raw.pdf]

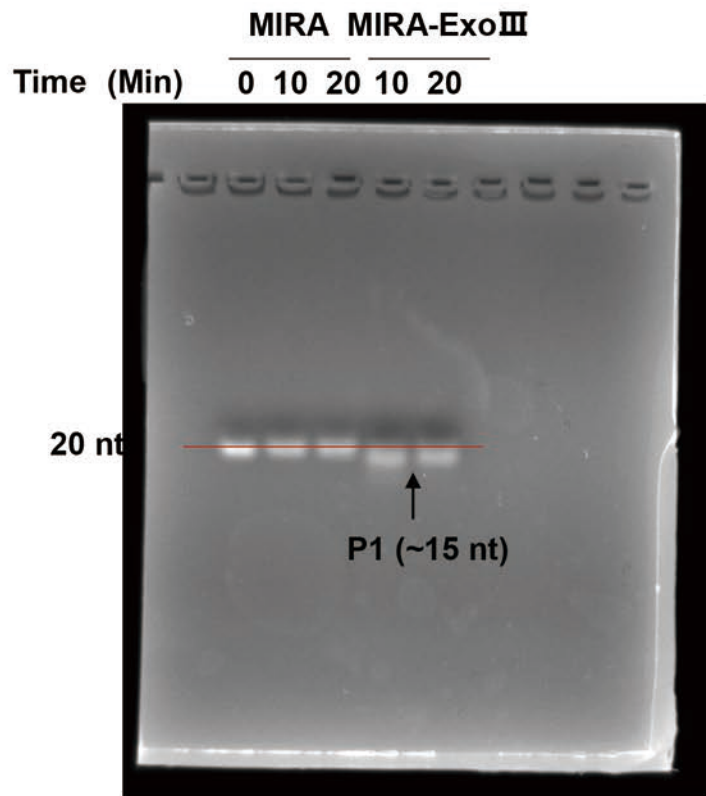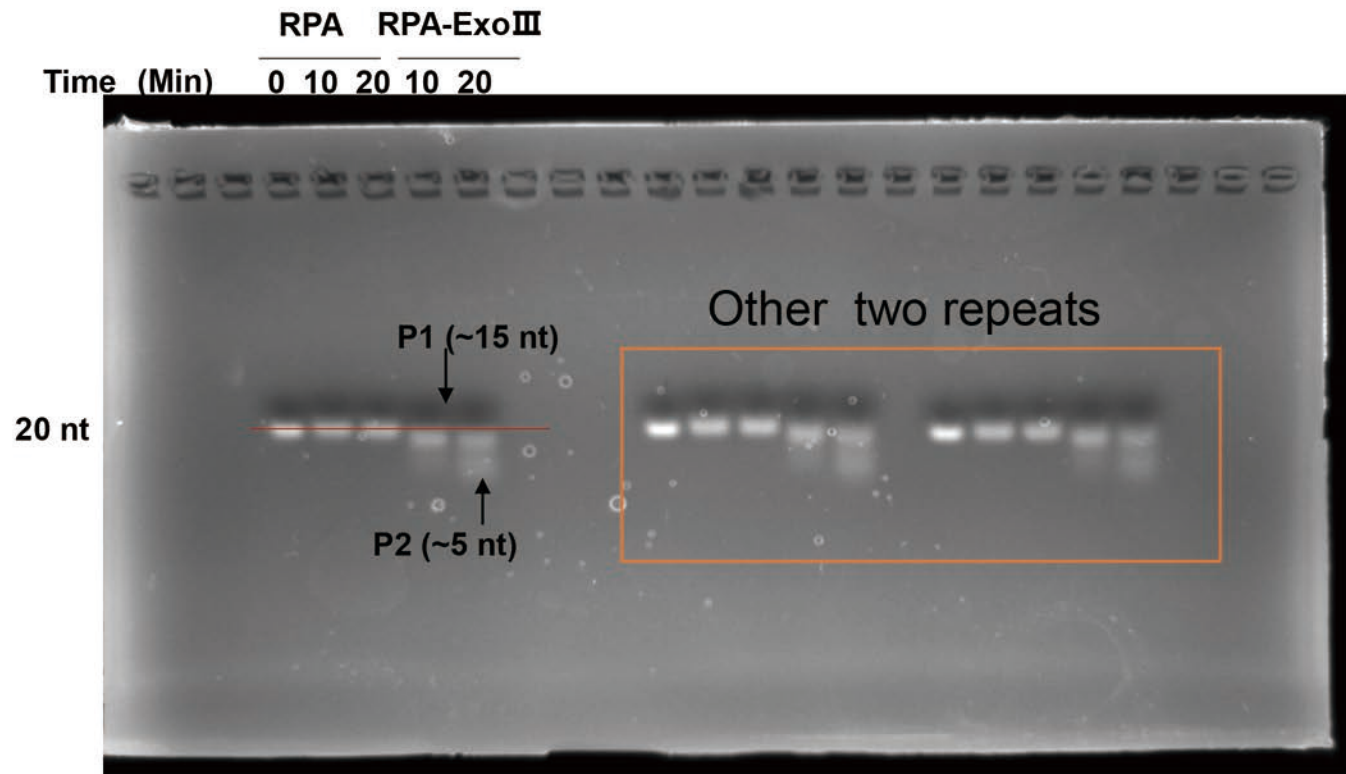

Supplement: Figure 5—source data 1. [file elife-95648-fig5-data1.zip › Figure 5-Source data/Figure 5C-labelled.pdf]

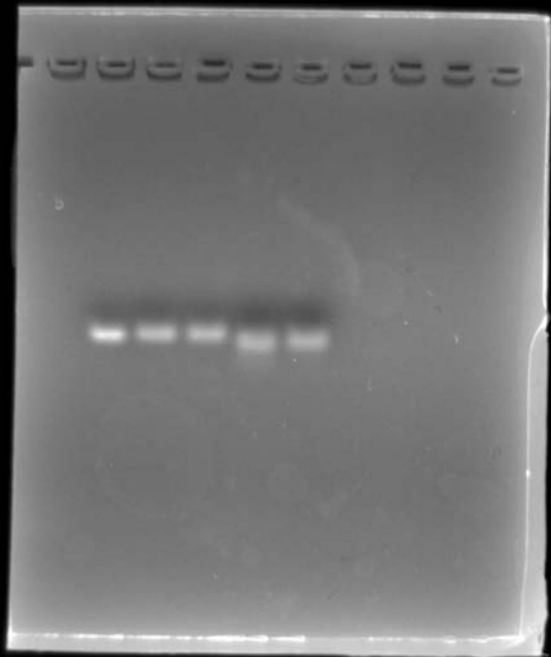

Supplement: Figure 5—source data 1. [file elife-95648-fig5-data1.zip › Figure 5-Source data/Figure 5C-left-raw.pdf]

**T4 gp32**  
**(10 mg/ml)**

**NT 0 1 5**

20 bp

5 bp

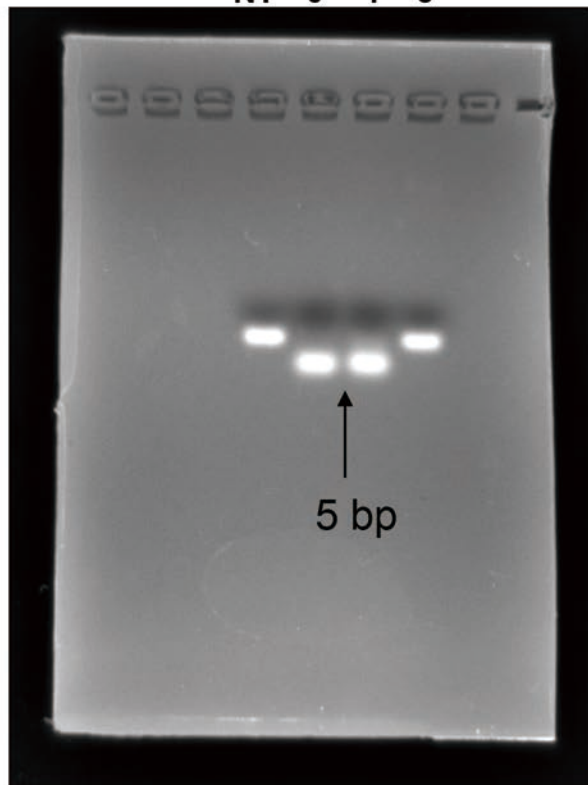

**SSB**  
**(1.58 mg/ml)**

**NT 0 1 5**

20 bp

5 bp

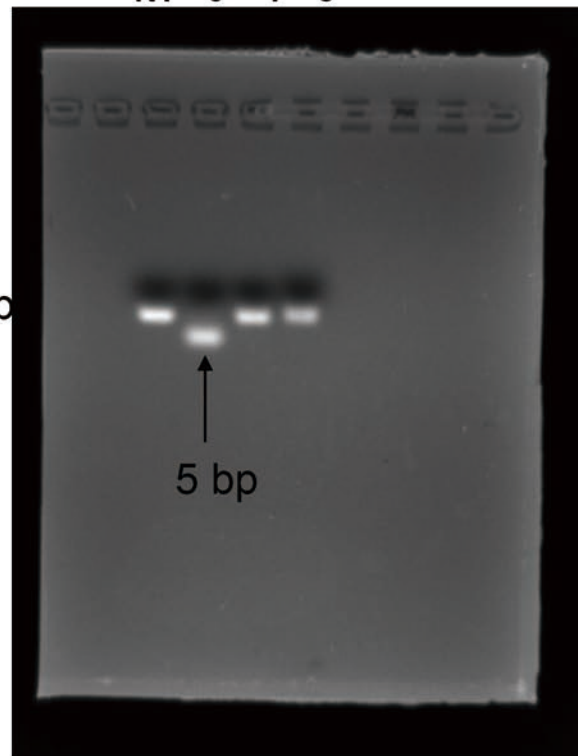

Supplement: Figure 5—source data 1. [file elife-95648-fig5-data1.zip › Figure 5-Source data/Figure 5F-labelled.pdf]

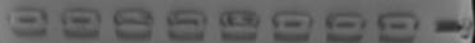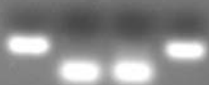

Supplement: Figure 5—source data 1. [file elife-95648-fig5-data1.zip › Figure 5-Source data/Figure 5F-left-raw.pdf]

S217A  
R216A  
W212A  
F213A  
F176A  
R170A  
K121A  
D214A  
Q112A  
N153A  
Y109A  
D151N  
Wild type

30 KD

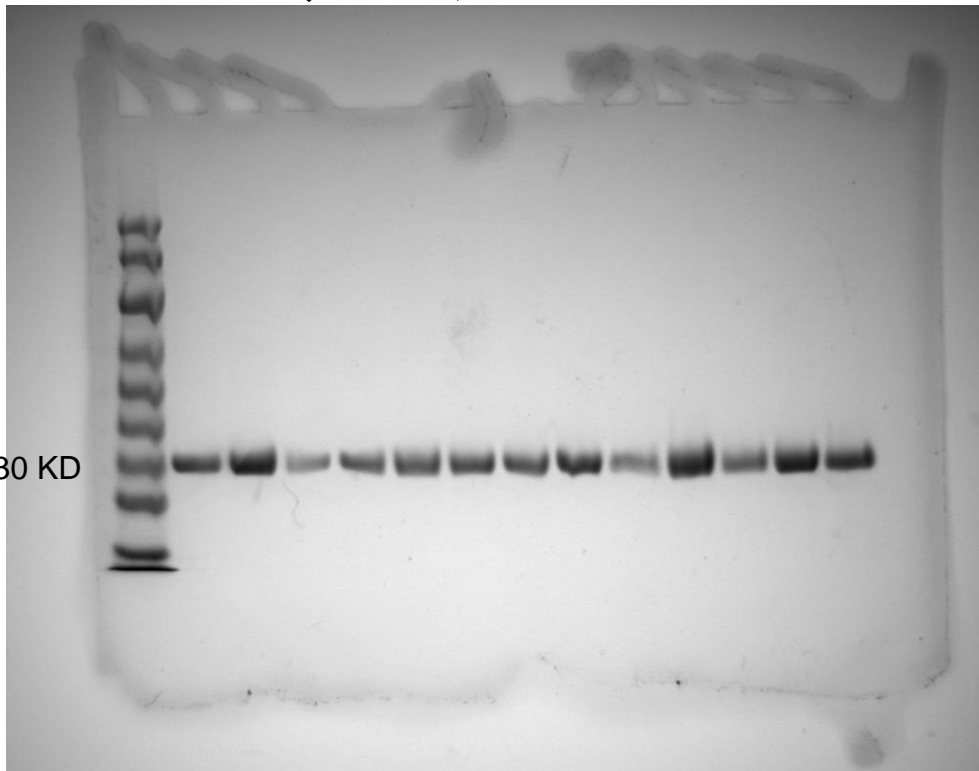

Supplement: Figure 6—source data 1. [file elife-95648-fig6-data1.zip › Figure 6-Source data/Figure 6A-labelled.pdf]

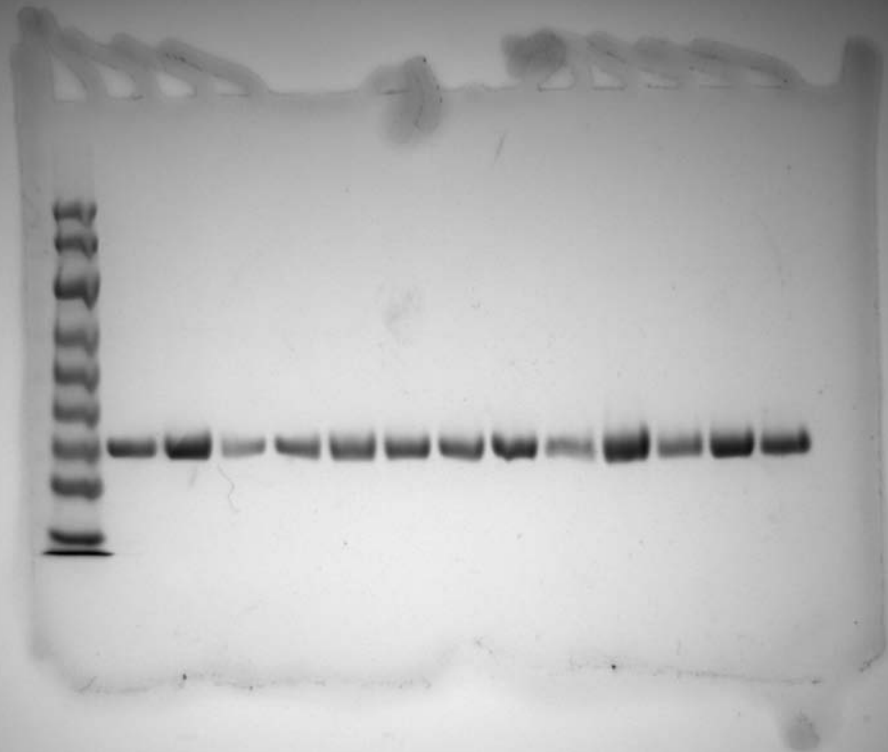

Supplement: Figure 6—source data 1. [file elife-95648-fig6-data1.zip › Figure 6-Source data/Figure 6A-raw.pdf]

# D151N

Time (Min) 0 0.5 1 2 5 10

20 nt

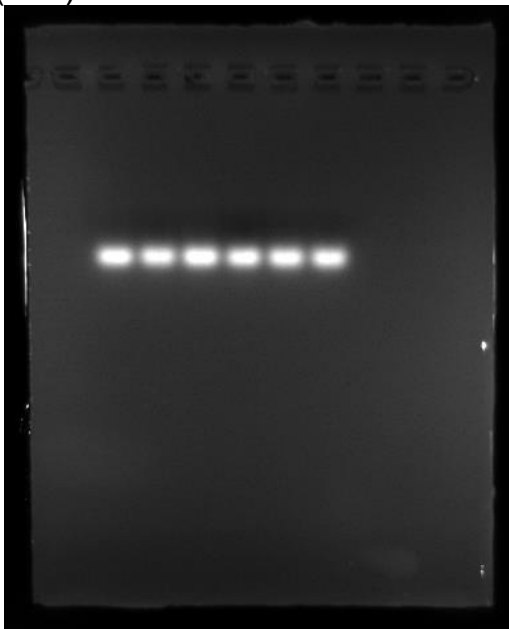

Supplement: Figure 6—source data 1. [file elife-95648-fig6-data1.zip › Figure 6-Source data/Figure 6C-D151N-labelled.pdf]

# D214A

Time (Min) 0 0.5 1 2 5 10

20 nt

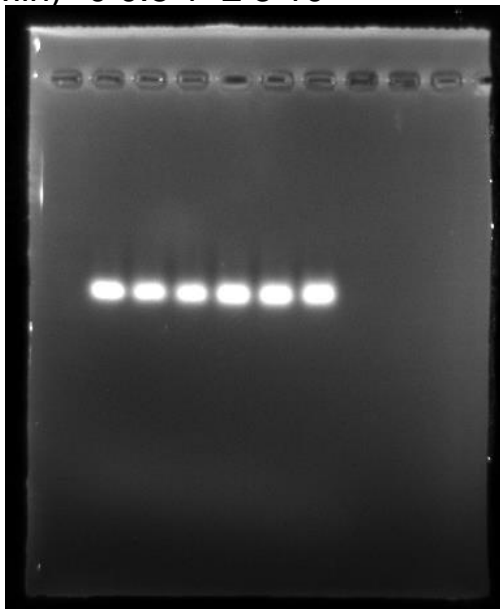

Supplement: Figure 6—source data 1. [file elife-95648-fig6-data1.zip › Figure 6-Source data/Figure 6C-D214A-labelled.pdf]

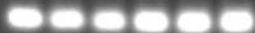

Supplement: Figure 6—source data 1. [file elife-95648-fig6-data1.zip › Figure 6-Source data/Figure 6C-D214A-raw.pdf]

# F213A

Time (Min) 0 0.5 1 2 5 10

20 nt

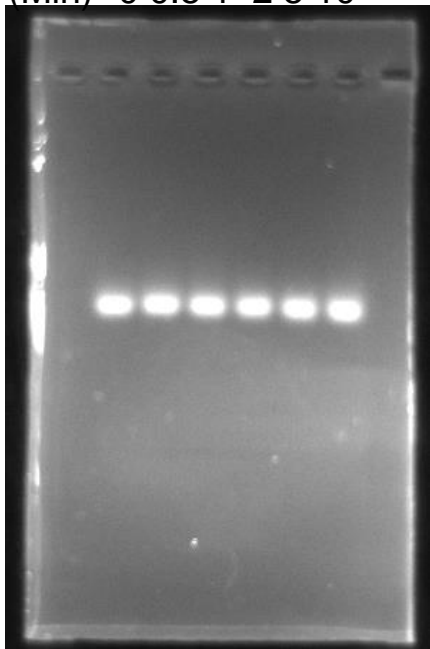

20 nt

Supplement: Figure 6—source data 1. [file elife-95648-fig6-data1.zip › Figure 6-Source data/Figure 6C-F213A-labelled.pdf]

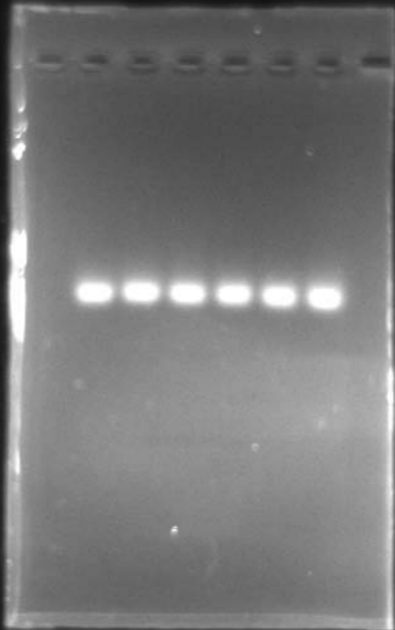

Supplement: Figure 6—source data 1. [file elife-95648-fig6-data1.zip › Figure 6-Source data/Figure 6C-F213A-raw.pdf]

# K121A

Time (Min) 0 0.5 1 2 5 10

20 nt

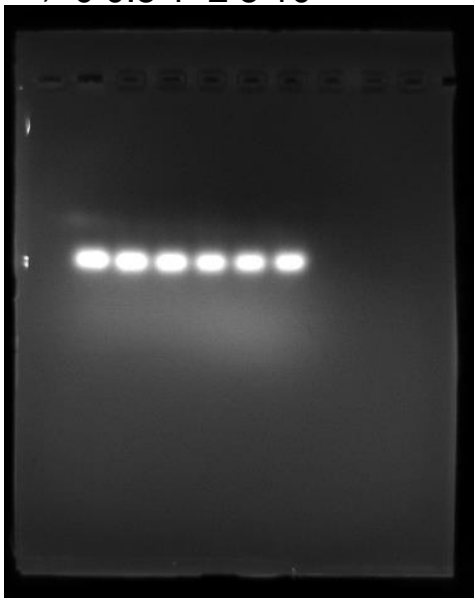

Supplement: Figure 6—source data 1. [file elife-95648-fig6-data1.zip › Figure 6-Source data/Figure 6C-K121A-labelled.pdf]

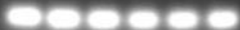

Supplement: Figure 6—source data 1. [file elife-95648-fig6-data1.zip › Figure 6-Source data/Figure 6C-K121A-raw.pdf]

# K176A

Time (Min) 0 0.5 1 2 5 10

20 nt

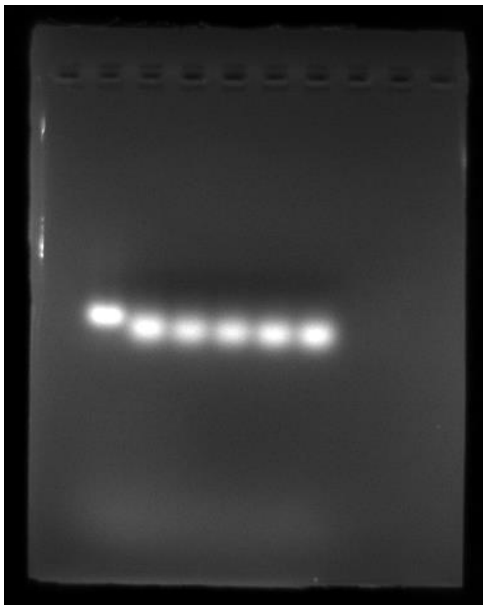

Supplement: Figure 6—source data 1. [file elife-95648-fig6-data1.zip › Figure 6-Source data/Figure 6C-K176A-labelled.pdf]

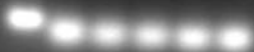

Supplement: Figure 6—source data 1. [file elife-95648-fig6-data1.zip › Figure 6-Source data/Figure 6C-K176A-raw.pdf]

# N153A

Time (Min) 0 0.5 1 2 5 10

20 nt

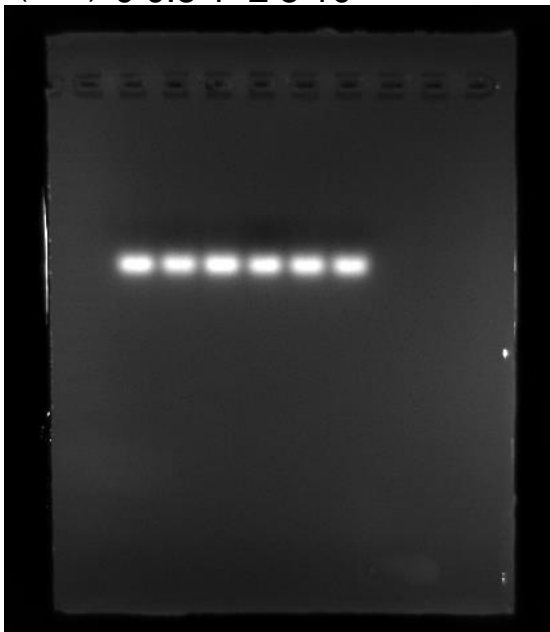

Supplement: Figure 6—source data 1. [file elife-95648-fig6-data1.zip › Figure 6-Source data/Figure 6C-N153A-labelled.pdf]

Q112A

Time (Min) 0 0.5 1 2 5 10

20 nt

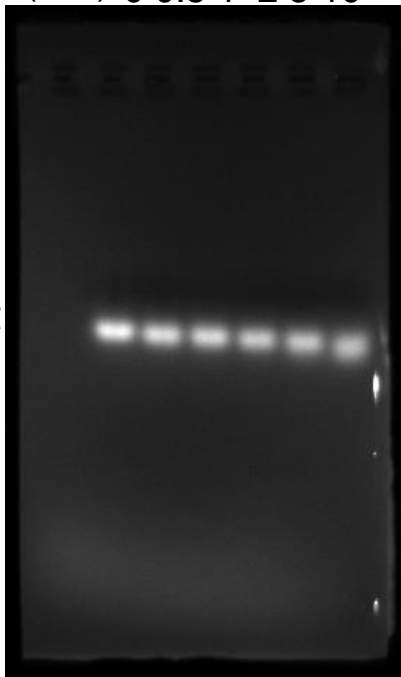

Supplement: Figure 6—source data 1. [file elife-95648-fig6-data1.zip › Figure 6-Source data/Figure 6C-Q112A-labelled.pdf]

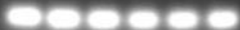

Supplement: Figure 6—source data 1. [file elife-95648-fig6-data1.zip › Figure 6-Source data/Figure 6C-Q112A-raw.pdf]

# R170A

Time (Min) 0 0.5 1 2 5 10

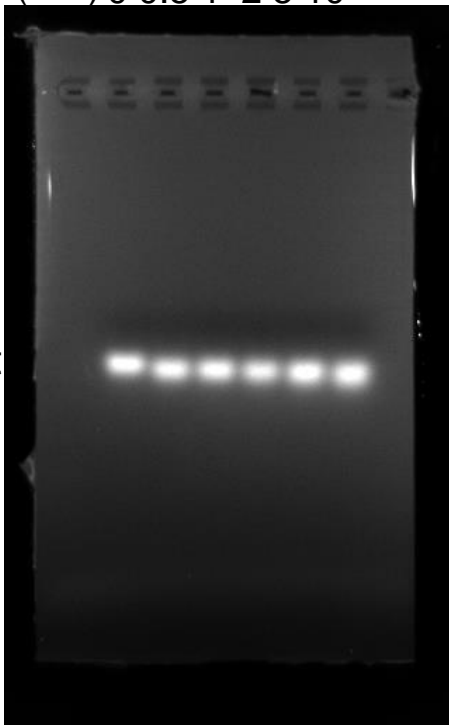

20 nt

Supplement: Figure 6—source data 1. [file elife-95648-fig6-data1.zip › Figure 6-Source data/Figure 6C-R170A-labelled.pdf]

# R216A

Time (Min) 0 0.5 1 2 5 10

20 nt

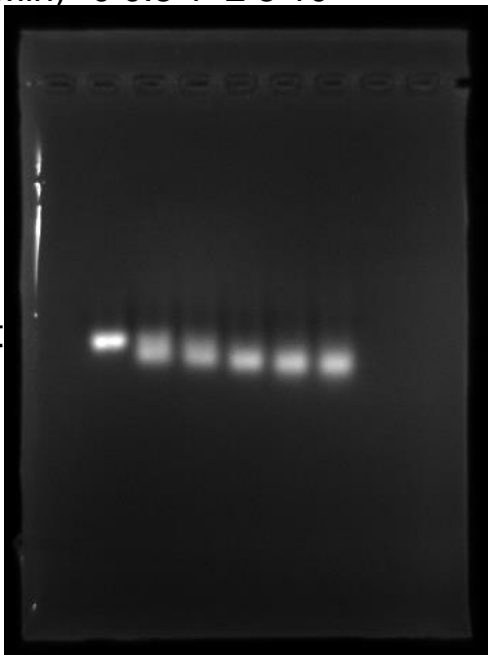

Supplement: Figure 6—source data 1. [file elife-95648-fig6-data1.zip › Figure 6-Source data/Figure 6C-R216A-labelled.pdf]

# S217A

Time (Min) 0 0.5 1 2 5 10

20 nt

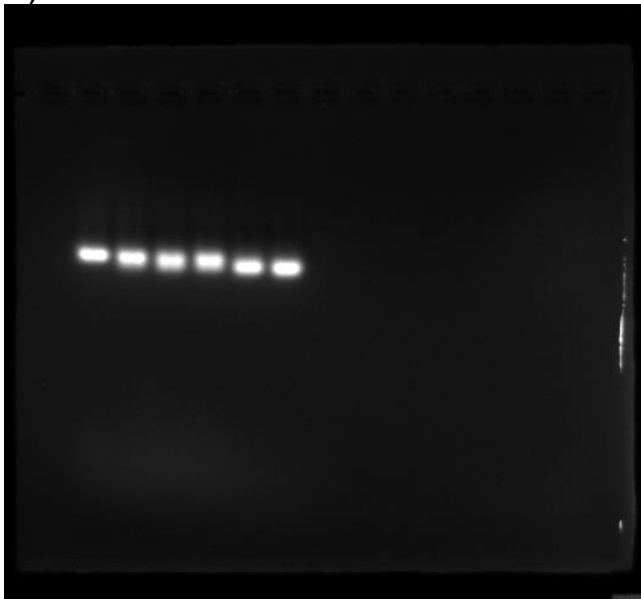

Supplement: Figure 6—source data 1. [file elife-95648-fig6-data1.zip › Figure 6-Source data/Figure 6C-S217A-labelled.pdf]

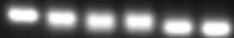

Supplement: Figure 6—source data 1. [file elife-95648-fig6-data1.zip › Figure 6-Source data/Figure 6C-S217A-raw.pdf]

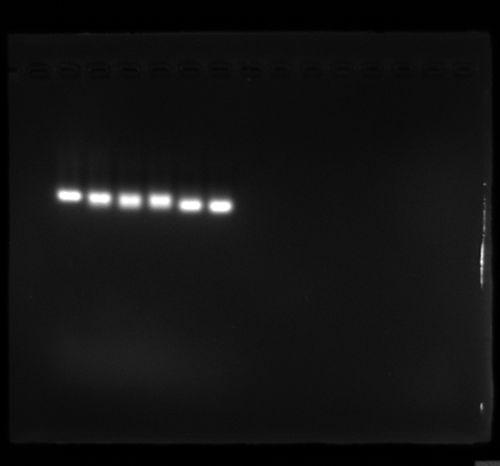

Supplement: Figure 6—source data 1. [file elife-95648-fig6-data1.zip › Figure 6-Source data/Figure 6C-S217A-raw.tif]

# W212A

Time (Min) 0 0.5 1 2 5 10

20 nt

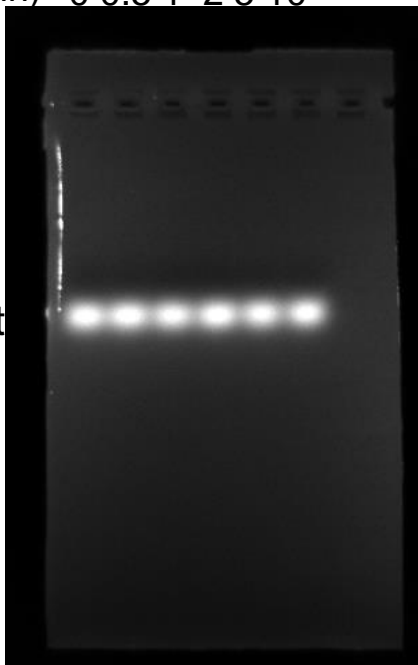

Supplement: Figure 6—source data 1. [file elife-95648-fig6-data1.zip › Figure 6-Source data/Figure 6C-W212A-lablelled.pdf]

WT

Time (Min) 0 0.5 1 2 5 10

20 nt

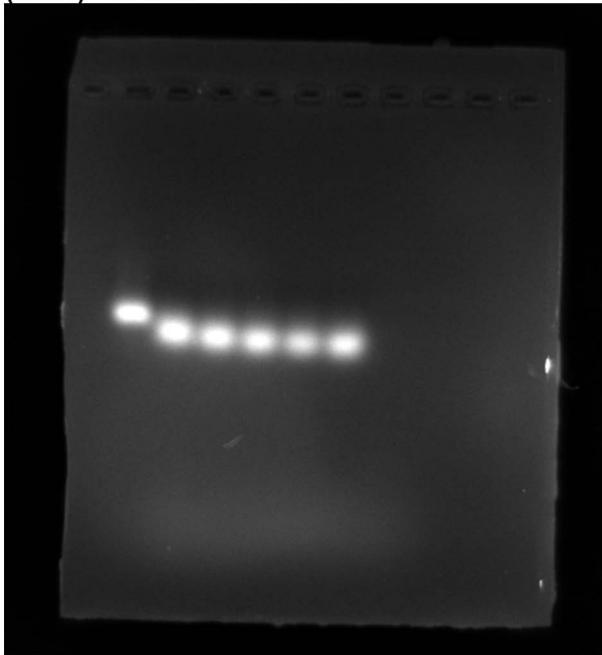

Supplement: Figure 6—source data 1. [file elife-95648-fig6-data1.zip › Figure 6-Source data/Figure 6C-WT-labelled.pdf]

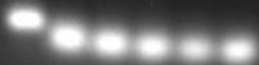

Supplement: Figure 6—source data 1. [file elife-95648-fig6-data1.zip › Figure 6-Source data/Figure 6C-WT-raw.pdf]

# Y109A

Time (Min) 0 0.5 1 2 5 10

20 nt

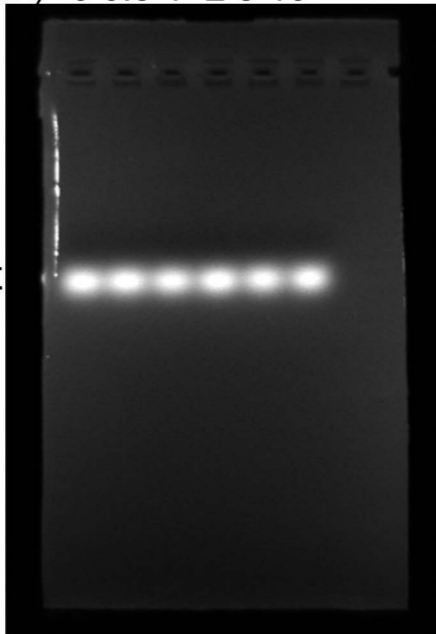

Supplement: Figure 6—source data 1. [file elife-95648-fig6-data1.zip › Figure 6-Source data/Figure 6C-Y109A-labelled.pdf]

M NT S217A R216A K176A Q112A Wild type R170A

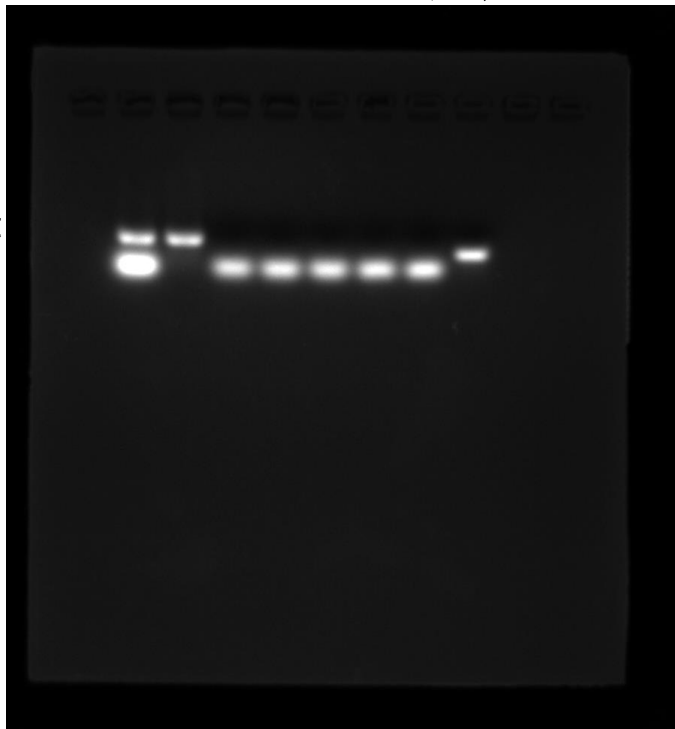

20 nt  
5 nt

Supplement: Figure 6—source data 1. [file elife-95648-fig6-data1.zip › Figure 6-Source data/Figure 6E-labelled.pdf]

1000 900 800 700 600 500 400 300 200 100 0

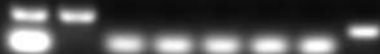

Supplement: Figure 6—source data 1. [file elife-95648-fig6-data1.zip › Figure 6-Source data/Figure 6E-raw.pdf]

S217A  
R216A  
W212A  
F213A  
F176A  
R170A  
K121A  
D214A  
Q112A  
N153A  
Y109A  
D151N  
Wild type

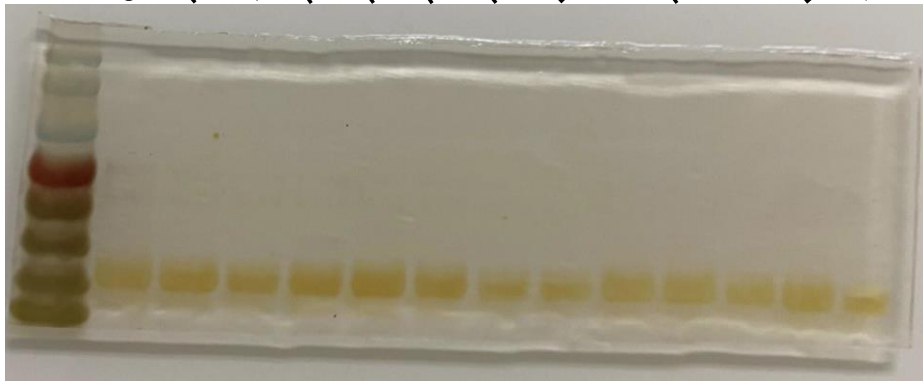

30 kD

Supplement: Figure 6—figure supplement 2—source data 1. [file elife-95648-fig6-figsupp2-data1.zip › Figure 6-figure supplement 2-Source data/Figure 6-figure supplement 2-labelled.pdf]

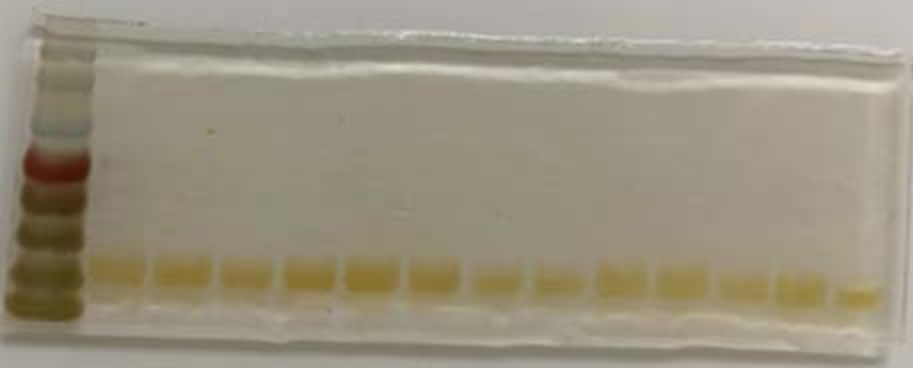

Supplement: Figure 6—figure supplement 2—source data 1. [file elife-95648-fig6-figsupp2-data1.zip › Figure 6-figure supplement 2-Source data/Figure 6-figure supplement 2-raw.pdf]

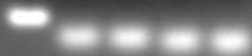

Supplement: Figure 6—figure supplement 3—source data 1. [file elife-95648-fig6-figsupp3-data1.zip › Figure 6-figure supplement 3-Source data/Figure 6-figure supplement 3D-left-raw.pdf]

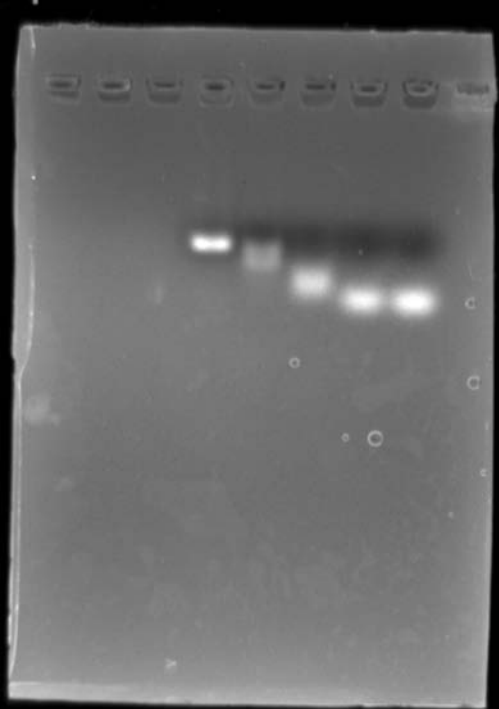

Supplement: Figure 6—figure supplement 3—source data 1. [file elife-95648-fig6-figsupp3-data1.zip › Figure 6-figure supplement 3-Source data/Figure 6-figure supplement 3D-right-raw.pdf]

## R170A

Time (Min) 0 1 2 5 10

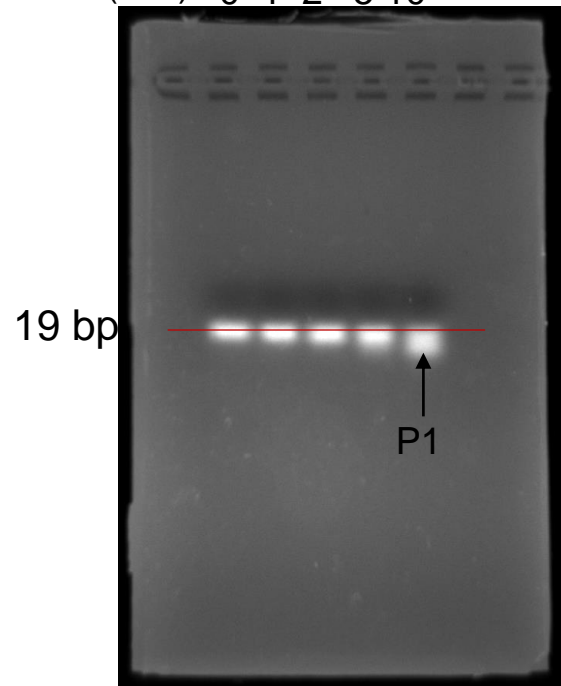

## Wild type

Time (Min) 0 1 2 5 10

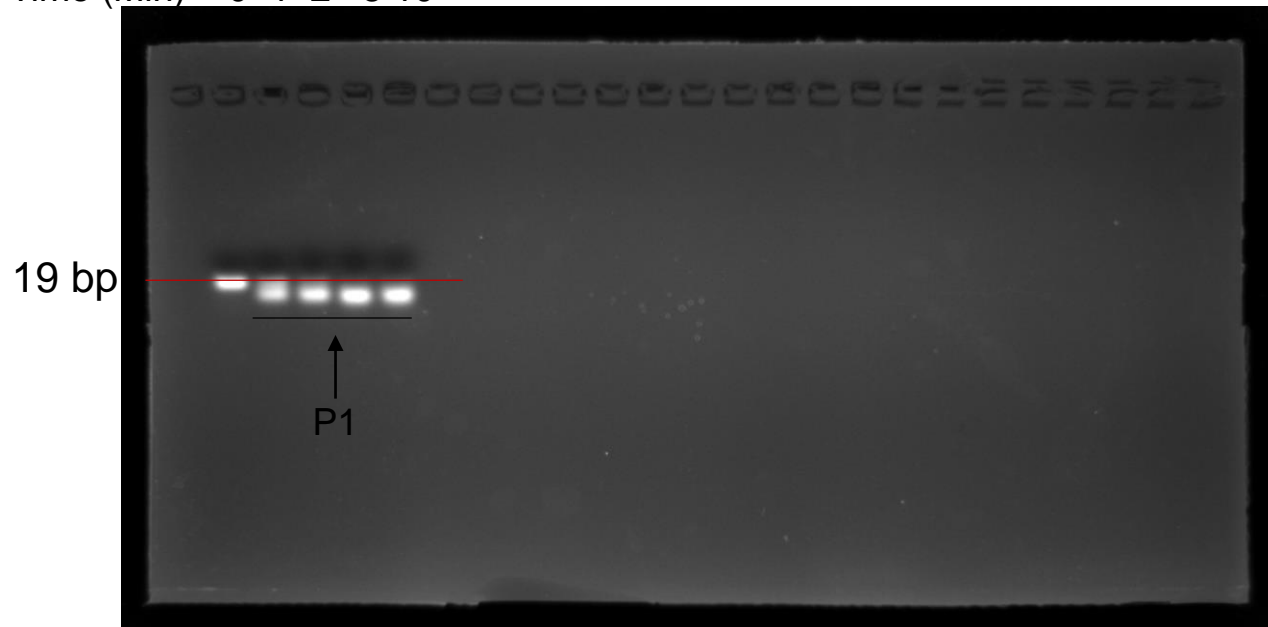

Supplement: Figure 6—figure supplement 3—source data 1. [file elife-95648-fig6-figsupp3-data1.zip › Figure 6-figure supplement 3-Source data/Figure 6¿Cfigure supplement 3A-labelled.pdf]

## R170A

Time (Min) 0 1 2 5 10

20 bp

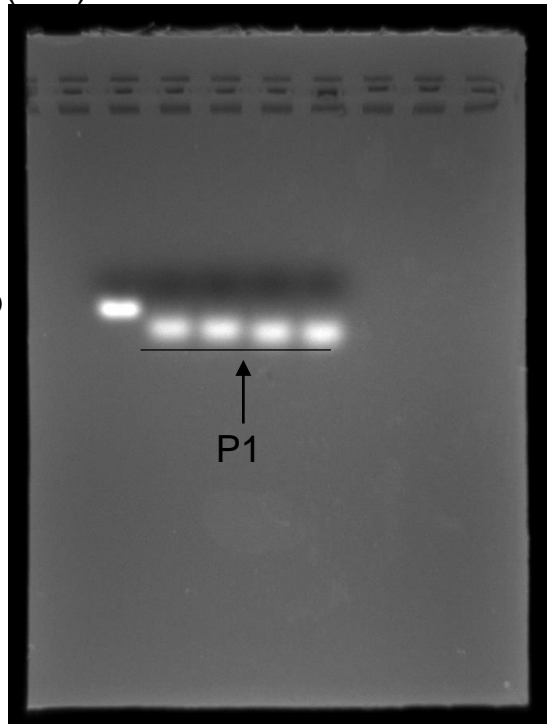

## Wild type

Time (Min) 0 1 2 5 10

20 bp

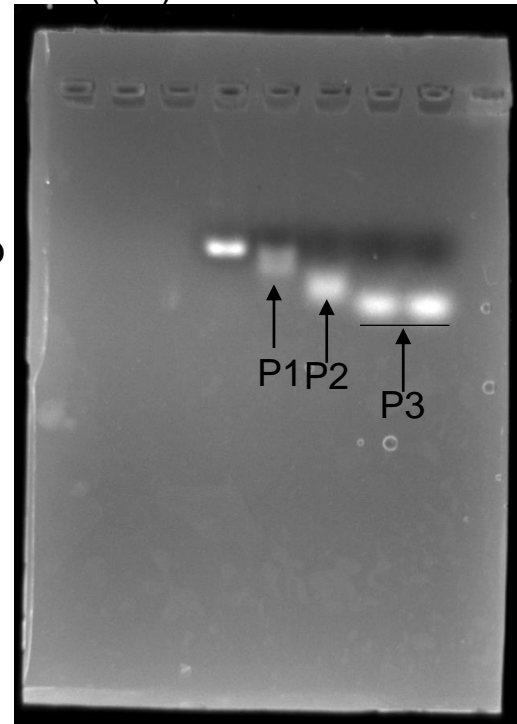

Supplement: Figure 6—figure supplement 3—source data 1. [file elife-95648-fig6-figsupp3-data1.zip › Figure 6-figure supplement 3-Source data/Figure 6¿Cfigure supplement 3D-labelled.pdf]

Time (Min)    0    1    2    5    10

5 nt

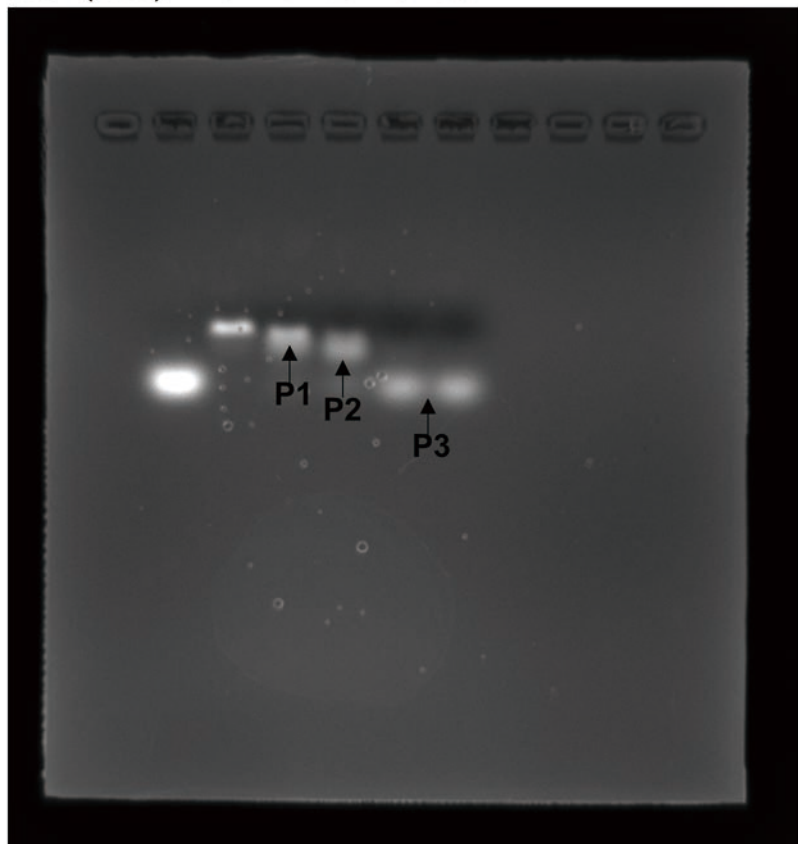

Supplement: Figure 8—source data 1. [file elife-95648-fig8-data1.zip › Figure 8-Source data/Figure 8A-labelled.pdf]

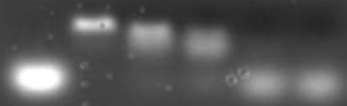

Supplement: Figure 8—source data 1. [file elife-95648-fig8-data1.zip › Figure 8-Source data/Figure 8A-raw.pdf]

# 10 base protruding

Time 0 0.5 1 2 10 (Min)

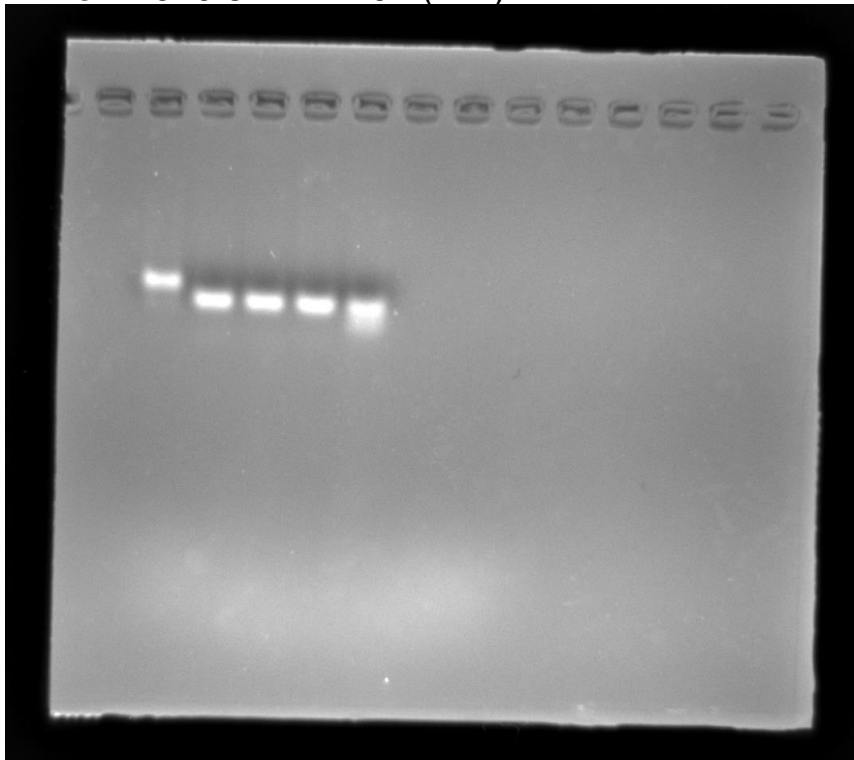

Supplement: Figure 8—figure supplement 1—source data 1. [file elife-95648-fig8-figsupp1-data1.zip › Figure 8-figure supplement 1-Source data/Figure 8-figure supplement 1A-labelled.pdf]

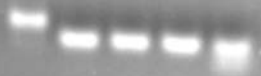

Supplement: Figure 8—figure supplement 1—source data 1. [file elife-95648-fig8-figsupp1-data1.zip › Figure 8-figure supplement 1-Source data/Figure 8-figure supplement 1A-raw.pdf]

# 4 base protruding

Time      0   0.5   1   2   10   (Min)

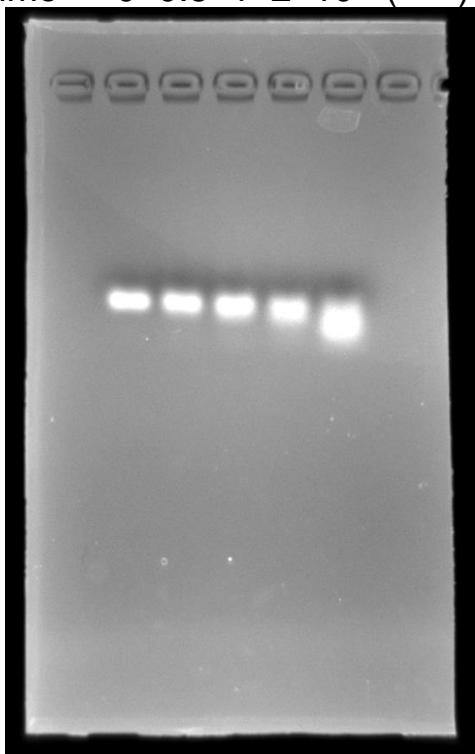

Supplement: Figure 8—figure supplement 1—source data 1. [file elife-95648-fig8-figsupp1-data1.zip › Figure 8-figure supplement 1-Source data/Figure 8-figure supplement 1C-labelled.pdf]

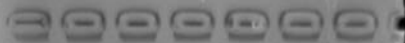

Supplement: Figure 8—figure supplement 1—source data 1. [file elife-95648-fig8-figsupp1-data1.zip › Figure 8-figure supplement 1-Source data/Figure 8-figure supplement 1C-raw.pdf]

## A4-protruding

A<sub>4</sub>-  
protruding  
-1 (23 bp)

A<sub>4</sub>-  
protruding  
-2 (20 bp)

Time 0 0.5 1 2 10 0 0.5 1 2 10 (Min)

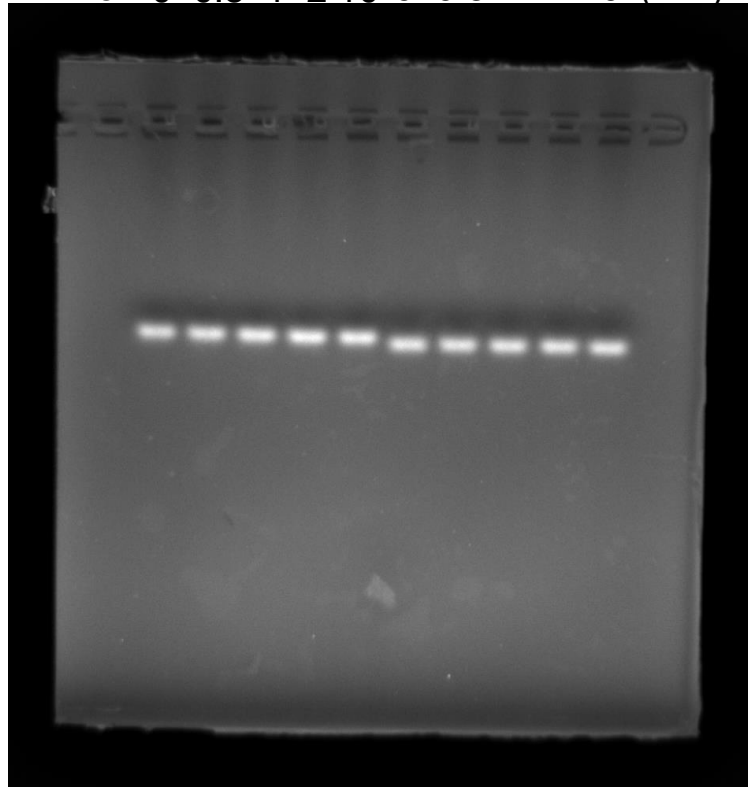

Supplement: Figure 8—figure supplement 1—source data 1. [file elife-95648-fig8-figsupp1-data1.zip › Figure 8-figure supplement 1-Source data/Figure 8-figure supplement 1E-labelled.pdf]

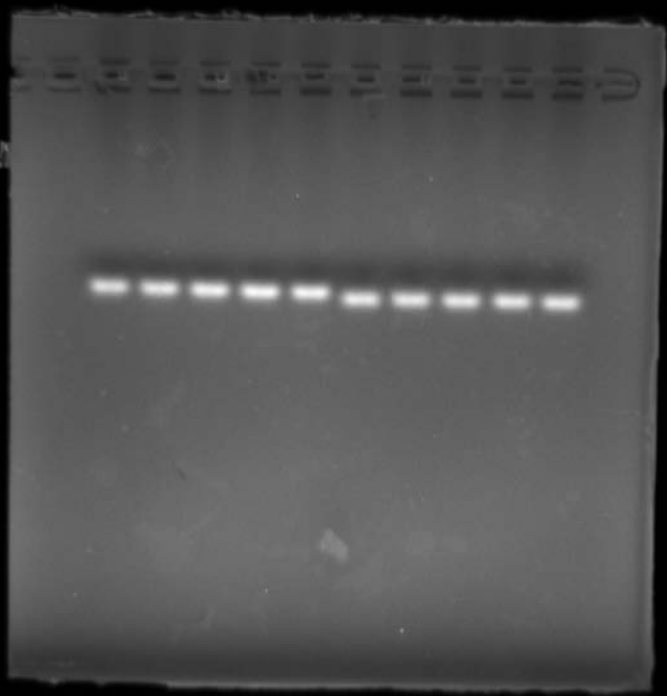

Supplement: Figure 8—figure supplement 1—source data 1. [file elife-95648-fig8-figsupp1-data1.zip › Figure 8-figure supplement 1-Source data/Figure 8-figure supplement 1E-raw.pdf]

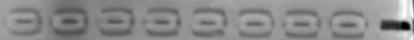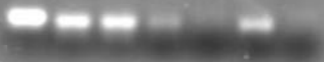

Supplement: Figure 8—figure supplement 1—source data 1. [file elife-95648-fig8-figsupp1-data1.zip › Figure 8-figure supplement 1-Source data/Figure 8-figure supplement 2A-raw.pdf]

T4 gp32 (10 mg/mL)

Time (Min)    0   1   2   5   10   1   0.2 (μl)

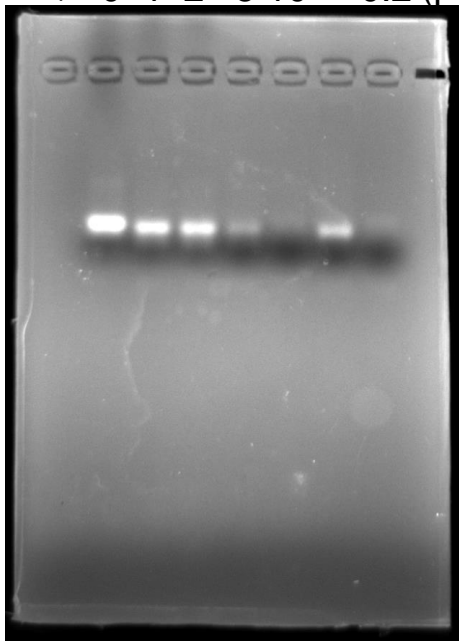

Supplement: Figure 8—figure supplement 1—source data 1. [file elife-95648-fig8-figsupp1-data1.zip › Figure 8-figure supplement 1-Source data/Figure 8¿Cfigure supplement 2A-labelled.pdf]

Time (Min) 0 1 2 5 10

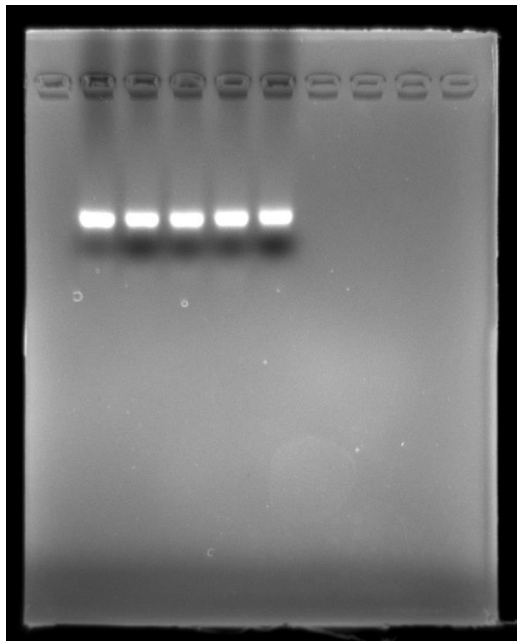

Supplement: Figure 8—figure supplement 1—source data 1. [file elife-95648-fig8-figsupp1-data1.zip › Figure 8-figure supplement 1-Source data/Figure 8¿Cfigure supplement 2E-labelled.pdf]
