# Supplementary material for: Bacterial exonuclease III expands its enzymatic activities on single-stranded DNA
Source: eLife. 2024 Jul 3;13:RP95648. doi: 10.7554/eLife.95648 (PMC11221836; doi:10.7554/eLife.95648)

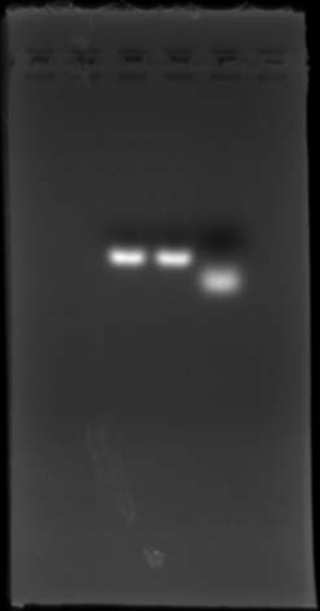

Supplement: Figure 4—source data 1. [file elife-95648-fig4-data1.zip › Figure 4/Figure 4B-Probe 3-raw.pdf]
