# Supplementary material for: Bacterial exonuclease III expands its enzymatic activities on single-stranded DNA
Source: eLife. 2024 Jul 3;13:RP95648. doi: 10.7554/eLife.95648 (PMC11221836; doi:10.7554/eLife.95648)

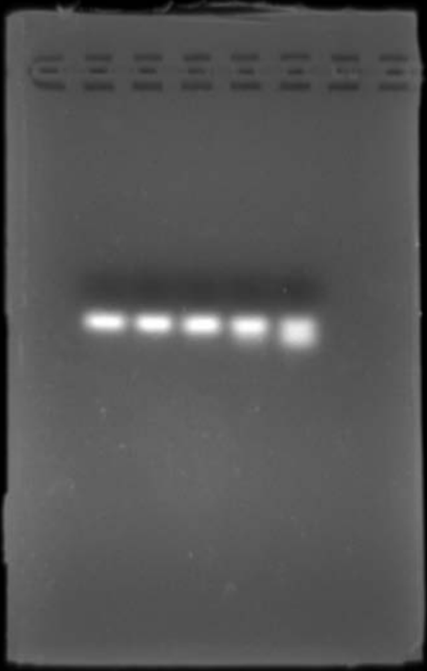

Supplement: Figure 6—figure supplement 3—source data 1. [file elife-95648-fig6-figsupp3-data1.zip › Figure 6-figure supplement 3-Source data/Figure 6-figure supplement 3A-left-raw.pdf]
