# Supplementary material for: Bacterial exonuclease III expands its enzymatic activities on single-stranded DNA
Source: eLife. 2024 Jul 3;13:RP95648. doi: 10.7554/eLife.95648 (PMC11221836; doi:10.7554/eLife.95648)
Supplement: Supplementary file 2. [file elife-95648-supp2.docx]

| **Supplementary File 2. A list of used sequences in the study.** | | | | |
| --- | --- | --- | --- | --- |
| Oligos | Sequences | 5′ Modifications | 3′ Modifications | Used |
| FQ reporter | TTATT | FAM (labeled at phosphonate) | BHQ1 (labeled at phosphonate) | Fig. 1, 4, 5, 6 |
| T_1_-labeled reporter | T_1_TATT | FAM (at T_1_ base) | BHQ1 (at phosphonate) | Fig. 1 |
| Base-labeled FQ reporter | TTATT | FAM (at T_1_ base) | BHQ1 (at T base) | Fig. 1 |
| Probe 1 | CAAACCCAGAGCCAATCTTATCT | FITC (at phosphonate) | None (-OH) | Fig. 2, 3, 4 |
| Probe 2 | GGGTGGGCGGAAAACTATTTC | FAM (at phosphonate) | None (-OH) | Fig. 2, 3, 4 |
| Probe 3 | AGTCCGTTTGTTCTTGTGGC | FAM (at phosphonate) | None (-OH) | Fig. 2, 3, 4, 5, 6 |
| Activator-S for Cas12a trans-cleavage activity | TTTCAACAGCACATGCAGAATCAT | None (-OH) | None (-OH) | Fig. 1, 2 |
| Activator-AS for Cas12a trans-cleavage activity | ATGATTCTGCATGTGCTGTTGAAA | None (-OH) | None (-OH) | Fig. 1, 2 |
| crRNA | GGUAAUUUCUACUAAGUGUAGAUAACAGCACAUGCAGAAUCAU | None (-OH) | None (-OH) | Fig. 1, 2 |
| A_20_ | AAAAAAAAAAAAAAAAAAAA | FAM (at phosphonate) | None (-OH) | Fig. 4 |
| C_20_ | CCCCCCCCCCCCCCCCCCCC | FAM (at phosphonate) | None (-OH) | Fig. 4 |
| T_20_ | TTTTTTTTTTTTTTTTTTTT | FAM (at phosphonate) | None (-OH) | Fig. 4 |
| Substrate-S for endonuclease activity | AAGATTGGCTCTGGGTTTGAAAA | FAM (at phosphonate) | None (-OH) | Fig. 6 |
| Substrate-AS for endonuclease activity | CAAACCCAG[THF]GCCAATCTTAAAA | None (-OH) | None (-OH) | Fig. 6 |
| Substrate-S for exonuclease activity | CAAACCCAGAGCCAATCTTATCT | FAM (at phosphonate) | None (-OH) | Fig. 6 |
| Substrate-AS for exonuclease activity | AGATAAGATTGGCTCTGGGTTTG | None (-OH) | None (-OH) | Fig. 6 |
| 10 base protruding structure-S | CAAACCCAGAGCCAATCTTATCT | FAM (at phosphonate) | None (-OH) | Fig. 8 |
| 10 base protruding structure-AS | GGCTCTGGGTTTGATCGATCGATCG | None (-OH) | None (-OH) | Fig. 8 |
| 4 base protruding structure | CAAACCCAGAGCCAATCTTATCT | FAM (at phosphonate) | None (-OH) | Fig. 8 |
| 4 base protruding structure-AS | AAGATTGGCTCTGGGTTTGATCG | None (-OH) | None (-OH) | Fig. 8 |
| A_4_ protruding structure-1-S | CAAACCCAGAGCCAATCTTAAAA | None (-OH) | None (-OH) | Fig. 8 |
| A_4_ protruding structure-1-AS | AAGATTGGCTCTGGGTTTGAAAA | None (-OH) | None (-OH) | Fig. 8 |
| A_4_ protruding structure-2-S | AGTCCGTTTGTTCTTGAAAA | None (-OH) | None (-OH) | Fig. 8 |
| A_4_ protruding structure-2-AS | CAAGAACAAACGGACTAAAA | None (-OH) | None (-OH) | Fig. 8 |
| Bubble-S | TGTGAGTTTTGAGCGTGGCGTGCTGGAGCAAAAA | None (-OH) | None (-OH) | Fig. 8 |
| Bubble-AS | TGCTCCAGCAATCGATCGATAAAACTCACAAAAA | None (-OH) | None (-OH) | Fig. 8 |
| Fork-S | TGTGAGTTTTGAGCGTGGCGTGCTGGAGCAAAAA | None (-OH) | None (-OH) | Fig. 8 |
| Fork-AS | TGCTCCAGCACGCCACGCTCATCGATCGAC | None (-OH) | None (-OH) | Fig. 8 |
| 3′flap on dsDNA-S-1 | AGTCCGTTTGTTCTTGTGGC | FAM (at phosphonate) | None (-OH) | Fig. 8 |
| 3′flap on dsDNA-S-2 | TAAGATTGGCAAAAA | None (-OH) | None (-OH) | Fig. 8 |
| 3′flap on dsDNA-AS | GCCAATCTTACAAACGGACTAAAAA | None (-OH) | None (-OH) | Fig. 8 |
| Note that S stands for sense strand, and AS stands for antisense strand. | | | | |
