# Supplementary material for: Bacterial exonuclease III expands its enzymatic activities on single-stranded DNA
Source: eLife. 2024 Jul 3;13:RP95648. doi: 10.7554/eLife.95648 (PMC11221836; doi:10.7554/eLife.95648)

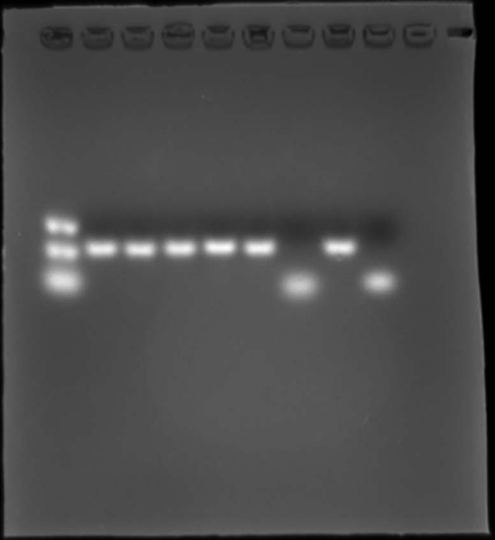

Supplement: Figure 2—source data 1. [file Figure 2B-raw.pdf › Figure 2-Source Data 1/Figure 2B-raw.pdf]
